# The effect of China’s new cooperative medical scheme on health expenditures among the rural elderly

**DOI:** 10.1186/s12939-019-0933-2

**Published:** 2019-02-06

**Authors:** Yanbing Zeng, Jiajing Li, Zhipeng Yuan, Ya Fang

**Affiliations:** 1 Key Laboratory of Health Technology Assessment of Fujian Province, School of Public Health, Xiamen University, Xiang’an Nan Road, Xiang’an District, Xiamen, 361102 Fujian China; 2 Center for Health Economics Experiment and Public Policy, School of Public Health, Shandong University, Jinan, China

**Keywords:** New cooperative medical scheme, Rural elderly, Health expenditures, Medical burden, Medical insurance

## Abstract

**Background:**

The alarming progression of an increasingly aging population in China has attracted much attention within the country and abroad. In 2003, the Chinese central government launched the New Cooperative Medical Scheme (NCMS) to resolve problems of healthcare inequity in regions with inadequate infrastructure and relative poverty. The purpose of this study was to investigate the effect of NCMS on health expenditures by the Chinese rural elderly population.

**Methods:**

The data were obtained from the Chinese Longitudinal Healthy Longevity Survey (CLHLS), which was conducted in 2005, 2008, 2011 and 2014. Elderly people living in rural areas and 60 years old or above were screened for the investigation. The sample size was 7472 in 2005, 11,705 in 2008, 9239 in 2011, and 6059 in 2014. The OOP% and reimbursement ratio were the medical expenses paid by individuals accounting for their per capita annual income and the medical expenses paid by medical insurance accounting for their total medical expenses, respectively. By controlling for individuals’ sociodemographic characteristics, pensions, demands and utilization of health services, we estimated the effect of the NCMS on the OOP% and reimbursement ratio for the rural elderly using seemingly unrelated regression (SUR).

**Results:**

The NCMS coverage ranged from 11.63% in 2005 to 80.34% in 2014. The medical expenses of the elderly also increased from an average of $204.77 in 2005 to $696.23 in 2014, which was more than three times as much as in 2005. From 2005 to 2014, the reimbursement ratio for medical expenses of rural elderly people with NCMS increased significantly from 30.6% in 2005 to 56.1% in 2014. The proportion of reimbursement ratio for rural seniors with NCMS increased by 6.4% across each survey cycle (every 3 years). However, the NCMS resulted in an insignificant decrease in OOP% by 1.4% across each survey cycle (every 3 years). Among other medical insurances, public insurance and private elder insurance had significant positive impacts on reimbursement ratio but did not influence OOP%.

**Conclusions:**

NCMS remarkably increased the rural elderly’s reimbursement ratio but insignificantly decreased the rural elderly’s OOP%. In addition, the proportion of reimbursement ratio for NCMS participants increased by 6.4% every 3 years. Lower outpatient reimbursement, migration, limited reimbursement scope, an increasing demand for medical services and the rapid growth of medical expenses may be reasons for the gaps between the nominal reimbursement ratio and the actual reimbursement ratio and OOP%. Policymakers should further modify NCMS policies in rural China.

## Background

China is facing a serious challenge regarding aging. According to the sixth national census survey in 2010, China had 178 million people more than 60 years old and 119 million more than 65 years old, which accounted for 13.3 and 8.9% of its total population [1], respectively. The health of the elderly worsens with age, and elderly people typically suffer from many chronic diseases [2]. Elderly patients have complicated social, nutritional, and dependency needs; consequently, the problem of their ever-increasing medical expenses has become prominent, and the burden of degenerative disease has been imposed on the agenda at all levels of government [3]. The National Bureau of Statistics in China showed that the annual growth rate of medical expenses for elderly people aged 65 years and above was 2.7% between 1990 and 2010 and was projected to be 2.2% between 2010 to 2030, which exceeds that of the US and other OECD member countries (0.3–0.5%).

The health of the rural elderly arouses great concern. The huge number of rural elderly and their deepening health problems (e.g., growing threats of infectious diseases and chronic diseases) place enormous pressure on health security for the elderly in rural China [4]. The subsidization of healthcare costs through insurance schemes is crucial in overcoming financial barriers to healthcare and avoiding high medical expenditures for patients in China [5]. In 2003, the Chinese central government launched a policy in rural areas called the ‘New Cooperative Medical System’ (NCMS). This scheme aims to safeguard farmers’ access to basic health services and alleviate the financial burden caused by sickness and poverty with a focus on inpatient services and catastrophic outpatient services [6]. Farmers were actively encouraged to enroll in the scheme as over 70% of the NCMS fund was contributed by the government and individual contributions were relatively small. The participants are entitled to reimbursements in designated hospitals and regulated by lower reimbursement rates in higher-level hospitals.

The NCMS has been further adjusted and optimized to reduce health expenditures by rural residents and achieve universal coverage, and even though personal insurance premiums have been increasing, the reimbursement scope and proportion are also gradually expanding. The annual premium per capita has gradually increased from ¥40 ($4.88) in 2005 to ¥400 ($65.11) in 2014. In 2005, the coverage of NCMS was 21% throughout the entire nation. Reimbursement coverage mainly involved serious diseases, and the nominal reimbursement ratio for hospitalization was approximately 30%. Outpatient expenditure coverage began in 2009. By the end of 2014, 736 million people had participated in the NCMS with a participation rate of 98.9%. At the same time, the upper limit of reimbursement was also raised, with outpatient reimbursement increasing to ¥80 ($13.02), outpatient observation reaching ¥1000 ($162.79), and total medical service reaching ¥200,000 ($32,557.38). In 2014, the nominal reimbursement ratio of hospitalization was 85% in township hospitals, 70% in county hospitals, 55% in municipal hospitals and 50% in provincial hospitals [5]. Through policy development of the NCMS, it is clear that the financing level and reimbursement of the NCMS have improved from 2005 to 2014. However, in 2011, the actual reimbursement ratio was far lower than the nominal reimbursement ratio of the NCMS plans [5].

The NCMS has been playing a growing and prominent role in alleviating the economic burden of farmers’ medical treatment [7]. Since the founding of the NCMS in 2003, several studies have been conducted to evaluate its effectiveness in providing financial protection to rural residents and reduce health inequalities. Most studies suggest that NCMS had substantially improved health care access and utilization among the participants but offered limited financial protection in terms of out-of-pocket (OOP) health spending and catastrophic expenditure risk [8–10]. For instance, Cheng et al.’s study shows that NCMS helped alleviate inequalities in terms of perceived access to health care among rural elderly but did not reduce out-of-pocket spending [11]. Liu et al. proved that the effects of NCMS expansion have been offset by the rapid escalation of medical expenditures [12]. OOP spending remains a severe burden for rural patients [8]. Some other empirical studies also found that NCMS participants had a slightly lower chance of facing catastrophic medical expenditures but the results were not statistically significant [13, 14].

However, with the change of the proportion and the reimbursement scope of NCMS, few studies have used panel data to prove that a reduction in out-of-pocket spending by the NCMS, which is income-related, would relieve the disease-related financial burden for patients [11]. It is necessary to reexamine previous findings about the influence of NCMS on participants’ health expenditures.

Therefore, this study aimed to 1) investigate the health expenditures and reimbursements of rural elderly people and 2) explore how the establishment and subsequent development of the NCMS influenced the medical financial burden of the Chinese rural elderly regarding OOP% and reimbursements.

## Methods

### Data sources

This study is a quantitative study based on microscopic data of the Chinese Longitudinal Healthy Longevity Survey (CLHLS).We focused on the impact of compensation policy changes on the medical economic burden of the rural elderly after the establishment of the NCMS. Therefore, we used four sets of data (2005, 2008, 2011 and 2014) from the CLHLS. The CLHLS is a large-scale, multidisciplinary, longitudinal follow-up survey of the elderly. The baseline survey started in 1998 and covered individuals aged 65 and above in 801 counties of 22 Chinese provinces and cities [15, 16]. Follow-up surveys of CLHLS were conducted in 2000, 2002, 2005, 2008–2009, 2011–2012, and 2014. Only the oldest (over 80 years) participants were surveyed before 2002, and health insurance coverage and healthcare expenditures were only asked since 2005 [11]; therefore, this study obtained samples from 2005 and subsequent waves for comparative analysis. Because we were concerned about the rural elderly, if the residential location was considered a rural area in the CLHLS four-wave data, then we included the data. The sample size was 7472 in the 2005 wave, 11,705 in the 2008–2009 wave, 9239 in the 2011–2012 wave, and 6059 in the 2014 wave.

### Variables

#### Dependent variables

OOP payments included all categories of health-related expenses paid directly by the household at the time the household received health services and typically included doctors’ consultation fees, medication purchases, and hospital bills [17]. However, insurance premiums were excluded because this study focused on measuring health expenditures directly related to illnesses. We calculated OOP% as the individual payment for medical services divided by their per capita annual income last year. In addition, we focused on the reimbursement ratio, which was the medical expenses paid by the medical insurances last year divided by their total medical expenses last year, including inpatient and outpatient costs. The item ‘Medical expenses paid by the family last year out of total medical costs’ in the CLHLS questionnaire indicates that medical expenses paid by respondents themselves in the last year relative to those reimbursed by insurance.

#### Independent variables

According to Grossman health demand theory [18, 19], we considered individuals’ socioeconomic characteristics, pensions and insurance and health service demand and utilization as independent variables. Basic personal information included gender, marital status, age, self-rated economic status, education, occupation before 60, family number, family total income in the past year. Health service demand and utilization included self-reported health, serious illnesses within the last 2 years, receipt of adequate medical service, medical service costs, and family expenses for medical services. Pension and insurance included retirement pension, public and free medical services, private or commercial elderly insurance, public elderly insurance, other social security or private insurance, the NCMS, and commercial medical insurance.

The variable of marital status was divided into the categories ‘currently married’ and ‘living with spouse’, and nonmarital status included the categories ‘divorced’, ‘widowed’ and ‘never married.’ The variable ‘serious illnesses within the last two years’ was changed into a dichotomous variable with ‘0’ and ‘1’, which represented the subject suffering from serious illness one or more times. ‘Occupation’ was a dummy variable that was ‘0’ for formal occupations, ‘1’ for self-employment and ‘2’ for no work. ‘Self-rated economic status’ and ‘self-reported health’ were ordered multicategorized variables in which ‘1’ to ‘5’ represented ‘very well’ to ‘very bad.’

Considering the time value of money, we multiplied the ‘medical service costs’, ‘medical service costs paid by family’ and ‘family total income’ of 2005, 2008, and 2011 by the inflation factor, which is 8% per year [20]. The exchange rate of the US dollar to RMB was 8.192 in 2005, 6.945 in 2008, 6.459 in 2011 and 6.143 in 2014.

### Analysis methods

In the past, researchers that performed empirical studies using more than two waves of the CLHLS usually deleted all censoring identification, which led to survivor bias [21]. This study was particularly focused on rural elderly people who spend a lot on medical care. These people are sensitive to price, and when in need of expensive healthcare they were likely to decline treatment, which results in shorter lives. Therefore, survivor bias would harm our results. To avoid survivor bias, this study selected IDs with two or more observations within the four waves, which constructed an unbalanced panel dataset.

We use a static two-equation seemingly unrelated regression (SUR) model framework for unbalanced panel data with latent individual heterogeneity and the reimbursement ratio and OOP% as endogenous variables [22–24]. The seemingly unrelated regressions (SUR) model explains the variation of not just one dependent variable, as in the univariate multiple regression model, but the variation of a set of many dependent variables [25]. SUR estimators performed consistently better than ordinary least squares (OLS) estimators since SUR takes the correlation between error terms into account [26].

All of the model versions we considered contained two equations, which had linear coefficients and were compactly written as y_git_ = xg_it_ + g_i_ + ug_it_, where g is the equation number (g = 1 represents reimbursement ratio and g = 2 represents OOP%), i is the ID number, and t is the number of periods in which the ID was observed.

The approach for this method is based on the construction of a multistep (stepwise) algorithm using the generalized least squares and maximum likelihood procedures. Based on this method, we built eq. 1 for OOP% endogenous variables and exogenous variables: the characteristics of sociology (except family number and total income), pension and insurance, and medical service demand and utilization (except family pay for medical services). We constructed eq. 2 for the following reimbursement ratio endogenous variables and exogenous variables: sociodemographic characteristics, pension and insurance, and medical service demand and utilization. Variables were filtered through the stepwise method to retain meaningful exogenous variables. All analyses were performed using Stata SE 15.1 (Stata Corp; College Station, TX).

## Results

The age, gender, marital status, occupation, degree of education and self-assessed economic status of Chinese rural elderly people between 2005 and 2014 are summarized in Table 1. In 2005, 81.94% of respondents reported that their economic status was so-so or better, and by 2014, that proportion rose to 87.20%. Hence, the household incomes per capita grew rapidly and self-rated economic status improved. The average age of the respondents increased from 81.11 to 84.99 years. The rural elderly who had serious illnesses once or more within the last 2 years increased from 16.31% in 2005 to 24.57% in 2014. The increase in the incidence of serious illness could at least partially be explained by the steady increase in mean age and aging structure of the population.

**Table 1:** Statistical description of demographic characteristics

Description	2005 (*N* = 7472)	2008 (*N* = 11,705)	2011 (*N* = 9239)	2014 (*N* = 6059)
	*n* (%)	*n* (%)	*n* (%)	*n* (%)
Age (year)	81.11 ± 10.78	84.14 ± 11.31	85.15 ± 11.17	84.99 ± 10.55
Family number	2.90 ± 1.89	2.75 ± 1.87	2.80 ± 2.14	2.43 ± 1.94
Gender				
male	2456 (45.04)	3930 (44.62)	3346 (45.24)	2369 (46.29)
female	2997 (54.96)	4877 (55.38)	4050 (54.76)	2749 (53.71)
Category of residence				
city and town	1065 (19.53)	1815 (20.61)	2698 (36.48)	2810 (54.90)
rural	4388 (80.47)	6992 (79.39)	4698 (63.52)	2308 (45.10)
Main occupation before age 60				
formal occupations	20 (0.37)	26 (0.30)	20 (0.27)	12 (0.26)
self-employment	5092 (93.64)	8285 (94.28)	6990 (94.91)	4324 (95.05)
no work	326 (6.00)	477 (5.43)	355 (4.82)	213 (4.68)
Current marital status				
nonmarital status	3128 (57.36)	5398 (61.29)	4464 (60.95)	2933 (58.88)
currently married	2325 (42.64)	3409 (38.71)	2860 (39.05)	2048 (41.12)
Education				
primary school	3411 (62.74)	5529 (62.94)	4513 (61.20)	2961 (57.98)
middle school and above	1364 (25.09)	2067 (23.53)	1751 (23.75)	2146 (42.02)
Self-rated economic status				
very rich	51 (0.94)	69 (0.79)	83 (1.14)	67 (1.35)
rich	746 (13.74)	979 (11.14)	1123 (15.43)	710 (14.26)
fair	3653 (67.26)	5986 (68.12)	4873 (66.96)	3566 (71.61)
poor	835 (15.38)	1449 (16.49)	969 (13.31)	521 (10.46)
very poor	146 (2.69)	305 (3.47)	230 (3.16)	116 (2.34)
Serious illness within the past two years				
0	4561 (83.69)	7465 (84.77)	5778 (79.53)	3650 (75.43)
1~	889 (16.31)	1341 (15.23)	1487 (20.47)	1189 (24.57)
Self-reported health				
very good	591 (11.25)	909 (11.24)	653 (9.47)	402 (8.51)
good	2205 (41.98)	3108 (38.43)	2422 (35.11)	1647 (34.86)
so-so	1710 (32.56)	2717 (33.60)	2607 (37.79)	1891 (40.02)
bad	690 (13.14)	1243 (15.37)	1119 (16.22)	694 (14.69)
very bad	56 (1.07)	110 (1.36)	98 (1.42)	91 (1.93)
Get adequate medical service				
yes	4772 (87.51)	8043 (91.33)	6832 (93.38)	4787 (96.01)
no	681 (12.49)	764 (8.68)	484 (6.62)	199 (3.99)
Retirement pension				
yes	475 (8.71)	677 (7.69)	629 (8.75)	510 (11.00)
no	4978 (91.29)	8130 (92.31)	6563 (91.25)	4126 (89.00)
Public free medical services				
yes	172 (3.15)	271 (3.08)	169 (2.35)	96 (2.08)
no	5281 (96.85)	8536 (96.92)	7038 (97.66)	4514 (97.92)
Private or commercial elder insurance				
yes	31 (0.57)	17 (0.19)	78 (1.08)	36 (0.78)
no	5422 (99.43)	8790 (99.81)	7128 (98.92)	4571 (99.22)
Public elder insurance				
yes	203 (3.72)	366 (4.16)	1316 (18.23)	1262 (27.11)
no	5250 (96.28)	8441 (95.84)	5904 (81.77)	3393 (72.89)
Other social security or private insurance				
yes	125 (82.78)	261 (2.97)	272 (4.15)	159 (3.78)
no	26 (17.22)	8541 (97.04)	6279 (95.85)	4053 (96.23)
NCMS				
yes	634 (11.63)	5340 (60.63)	5767 (79.10)	3947 (80.34)
no	4819 (88.37)	3467 (39.37)	1524 (20.90)	966 (19.66)
Commercial medical insurance				
yes	52 (0.95)	29 (0.33)	25 (0.35)	46 (1.00)
no	5401 (99.05)	8778 (99.67)	7176 (99.65)	4571 (99.00)

Fortunately, and with the development of primary healthcare, the proportion of patients who did not receive timely medical care decreased to only 3.99%. However, the health status of rural elderly also declined over the past decade. In 2005, more than half of the elderly believed they were in good or very good health, which was down to approximately 43% in 2014. Self-reported health and the number of serious diseases reflect the medical service demand of rural elderly people. As descriptive statistics of pensions and insurance demonstrates, the number of rural elderly with public elderly insurance and NCMS increased dramatically between 2005 and 2014. The NCMS coverage increased from 11.63% in 2005 to 80.34% in 2014. The coverage of public elder insurance expanded from 3.72% in 2005 to 27.1% in 2014 but other forms of pensions and insurance did not significantly change.

Table 2 shows the medical service cost and reimbursement ratio for the rural elderly. The average number of family members decreased while the total household incomes increased sharply from $895.26 in 2005 to $4541.92 in 2014. In addition, the medical expenses of the elderly also increased from an average of $204.77 in 2005 to $696.23 in 2014, which was more than three times as much as in 2005. Thus, even with the rapid promotion of medical service costs the total household income was growing faster. The reimbursement ratio for the medical expenses of rural elderly people with NCMS increased significantly from 23.6% in 2005 to 56.1% in 2014. OOP% remained at approximately 20% during the 10 years.

**Table 2:** Medical expenses and the reimbursement of rural elderly with NCMS

DESCRIPTION	Mean ± SD			
	2005	2008	2011	2014
Medical service costs in total ($)	204.77 ± 588.57	271.81 ± 921.35	510.37 ± 1727.389	696.23 ± 1866.99
Personal pay for medical services ($)	185.53 ± 551.64	226.99 ± 765.34	359.20 ± 1068.68	177.81 ± 534.99
Family total income ($)	895.26 ± 1642.11	2972.74 ± 3449.97	4196.31 ± 4752.87	4541.92 ± 4693.29
OOP% (%)	18.2 ± 29.1	26.1 ± 33.6	25.7 ± 36.6	17.6 ± 28.7
Reimbursement ratio (%)	23.6 ± 41.3	24.9 ± 40.4	43.2 ± 44.5	56.1 ± 42.1

Table 3 presents the influencing factors of OOP% and reimbursement ratio selected by the SUR model. In 2005, the variable ‘other social security or private insurance’ was more than 90% missing and therefore wasn’t included in the regression equation. As the result of the OOP% equation showed, the regression coefficient of the NCMS was positive but not significant, which indicated that the OOP% was reduced slightly by NCMS. Among the sociodemographic characteristic variables, rural elderly people who were married had a poor self-rated economic status and formal occupation before 60 had a higher OOP%. Among medical service demand and utilization variables, rural elderly with a higher OOP% suffered from poor health status and serious illnesses in the last 2 years and spent a lot on medical services. The higher OOP% usually did not have retirement wages or pensions.

**Table 3:** Influence factors of OOP% and reimbursement by SUR

	Coef.	Sd	z	P > |z|
OOP%				
Residence	0.062	0.008	7.600	0.000
Gender	0.006	0.009	0.730	0.464
Marriage	0.041	0.009	4.280	0.000
Age	0.000	0.000	−0.700	0.486
Economics	0.064	0.006	10.920	0.000
Education	−0.010	0.006	−1.480	0.140
Occupation 1 (refer to formal occupations)	−0.196	0.045	−4.360	0.000
Occupation 2 (refer to formal occupations)	−0.221	0.049	−4.520	0.000
Retirement wage	−0.066	0.015	−4.340	0.000
Pension	−0.046	0.011	−4.130	0.000
Public insurance	−0.056	0.032	−1.730	0.083
Commercial medical insurance	0.003	0.067	0.040	0.968
NCMS	−0.014	0.009	−1.540	0.124
Private elder insurance	−0.015	0.049	−0.300	0.761
Self-reported health	0.046	0.004	10.590	0.000
Serious illness times	0.187	0.011	17.450	0.000
Get adequate medical service	0.005	0.014	0.360	0.719
Medical service costs	0.000	0.000	17.370	0.000
Reimbursement ratio				
Residence	−0.073	0.006	−12.250	0.000
Gender	−0.022	0.006	−3.410	0.001
Marriage	0.024	0.007	3.550	0.000
Age	0.002	0.000	8.260	0.000
Economics	−0.020	0.004	−4.490	0.000
Education	0.013	0.005	2.800	0.005
Occupation 1 (refer to formal occupations)	0.434	0.033	13.310	0.000
Occupation 2 (refer to formal occupations)	0.391	0.035	11.050	0.000
Family number	−0.009	0.002	−5.550	0.000
Family total income	0.000	0.000	−1.040	0.296
Retirement wage	0.070	0.011	6.280	0.000
Pension	0.076	0.008	9.420	0.000
Public insurance	0.171	0.023	7.290	0.000
Commercial medical insurance	0.063	0.049	1.290	0.196
NCMS	0.064	0.006	9.910	0.000
Private elder insurance	0.114	0.036	3.200	0.001
Self-reported health	−0.023	0.003	−7.300	0.000
Serious illness times	−0.019	0.007	−2.610	0.009
Get adequate medical service	−0.068	0.010	−6.920	0.000

According to the second part of Table 3, the NCMS could significantly increase the reimbursement ratio. Among rural old people, widowed or divorced younger elderly women with poor economic conditions, a low educational level, formal occupation before 60, and a larger family had a lower reimbursement ratio. Formal occupations in this study included technicians/doctors/teachers, managers, general staff/servicer/workers, and military. Compared to freelancers, farmers, domestic workers or the unemployed, the reimbursement ratio of formal professionals was lower and the OOP% was higher. The proportion of reimbursement ratio for rural seniors with NCMS increased by 6.4% across each survey cycle (every 3 years). The proportion of reimbursement for the elderly without retirement wages, pensions, public medical insurance, NCMS and private medical insurance was lower. In addition, rural seniors whose self-report health status was poor suffered from serious illness last year and could get adequate medical service with a higher OOP%.

## Discussion

Since the founding of the NCMS in 2003, personal insurance premiums have been increasing and the reimbursement scope and proportion are also gradually expanding. This study mainly focused on the developing period of the NCMS. We found that the NCMS coverage was 11.6% in 2005 and increased to 80.3% in 2014. With the promotion of coverage and the change of insurance, the NCMS could effectively and significantly elevate the actual reimbursement ratio of the medical financial burden of Chinese rural seniors. This finding is in line with an existing study by Wang et al. [27]. From 2005 to 2014, the reimbursement ratio for medical expenses of rural elderly people with NCMS increased significantly from 30.6% in 2005 to 56.1% in 2014. In addition, the proportion of reimbursement ratio for rural seniors with NCMS increased by 6.4% across each survey cycle (every 3 years). However, the NCMS resulted in an insignificant decrease in OOP% by 1.4% across each survey cycle (every 3 years). Among other medical insurances, public insurance and private elder insurance had significant positive impacts on reimbursement ratio but did not influence OOP%. The innovation of this study was that it considered truncated data and effectively avoided survivor bias.

In 2014, the NCMS stipulated that the reimbursement ratio varied from 50% at provincial hospitals to 85% at township hospitals. According to the results of this study, the actual reimbursement ratio of the rural elderly with the NCMS as the main medical insurance was only 56% in 2014, which was the highest proportion in the past decade. Previous studies also found that effective reimbursement ratio of both outpatient and inpatient services were much lower than nominal reimbursement ratio that were originally designed by NCMS plans [5].

There may be several reasons for the gap between the nominal reimbursement ratio and the actual reimbursement ratio and OOP%, which was not significantly decreased by the NCMS. First, the low reimbursement ratio of the outpatient service in the NCMS was one of the reasons why the medical burden of the rural elderly has not been reduced. Of the total available NCMS funds, 70% were allocated to inpatient reimbursements and 30% to outpatient services [28]. The NCMS set an upper limit for outpatient reimbursement. These regulations made farmers’ benefits less extensive. An early empirical study also found that medical expenditures for outpatient services significantly increased for chronic disease patients [12]. Among people who had at least one outpatient visit during the past month, people with NCMS tend to have the highest OOP payments [29].

Second, during the 10 years of the survey, some respondents migrated from rural areas to cities. Of the respondents we screened, 19.53% lived in cities in 2005, which rose to 54.9% by 2014. The NCMS financing did not achieve national unification and therefore migrants may not be able to obtain reimbursement for medical treatment in other places, which results in a higher OOP% of their own medical expenditures. A previous study regarding the impact of NCMS on migrants showed that 55.2% of migrants in comparison with 24.6% of nonmigrants received no reimbursement from the NCMS [30]. Until the beginning of 2017, when the National Health Planning Commission issued a NCMS cross-provincial settlement reimbursement policy, this problem was unresolved. However, the effect of the solution remains to be verified.

Third, due to the low level of financing of NCMS, the limited compensation scope of NCMS may also be a reason. Ma et al. also found that a limited benefit package excluding many drugs or medical checks mainly resulted in high OOP spending and a considerable gap between the effective reimbursement ratio and the nominal reimbursement ratio [5]. The list of basic drugs was limited, and some expensive new drugs were not in the expense account. Injury has become the second leading cause of death in China, but the NCMS reimbursement excludes some accidental injuries, which indicates that these patients must pay for themselves. In recent years, new medical technology gradually entered the hospital. The NCMS only reimburses 200 yuan (at most) for Computed tomography (CT), Magnetic resonance imaging (MRI) and other auxiliary examinations, which makes the insured more self-sufficient.

Finally, an increasing demand for medical services and the rapid growth of medical expenses may also be an important reason. According to our results, the medical expenses of the rural elderly increased from an average of $204.77 in 2005 to $696.23 in 2014, which was more than three times as much as in 2005. The effects of the NCMS expansion have been offset by the rapid escalation of medical expenditures [12]. From 2005 to 2014, the self-assessed health level of the elderly in rural China gradually declined, which is possibly associated with higher social expectations. In addition, the number of serious diseases increased; therefore, the demand for medical services continued to increase. As the results of the regression model of OOP% and reimbursement ratio showed, rural elderly with poor health and serious illness in the past year had higher OOP% and a lower reimbursement ratio.

Therefore, we suggest that future NCMS policy revisions should maintain reimbursement ratio at their current levels. We can only effectively reduce the proportion of self-payments and the medical financial burdens of rural elderly people by controlling medical expenses. The financial protection of NCMS with average reimbursements of 56.1% in 2014 was modest and should be restructured to provide better benefits targeted for those with the most need [31].

According to the regression results of the two endogenous variables, rural elderly people with relatively poor economic conditions and poor health levels have higher OOPs and lower reimbursement ratio. Poor and unhealthy rural elderly people are at high medical financial risk. A study by Lai et al. also found that the benefits of NCMS are concentrated toward economically affluent groups [32]. Unreasonably increasing health expenses have aggravated the medical financial burden of low-income households [5]. In the NCMS, the pro-rich inequality dominated for inpatient care, while there was also a pro-poor advantage [33]. Rural people who haven’t benefitted from China’s economic development may not benefit from the recent healthcare reform and finance mechanisms unless schemes, such as the NCMS, provide more substantial subsidies [34]. The impact of NCMS in demoting medical service expenses of rural residents is still limited. The government should set up corresponding medical assistance to prevent poverty in these people.

There were some limitations in this study. First, with the rapid growth of NCMS coverage, we were unable to compare the medical expenses of elderly people with or without NCMS and failed to identify mutual factors influencing OOP% for all rural elderly people. Second, changes in health insurance for migrants from rural to urban areas have not been included. For instance, in 2014, some interviewees failed to report their hospitalization expense reimbursements to CLHLS in time, which may lead to lower OOP% and higher reimbursement ratio than the actual situation. Therefore, the financial protection effect of NCMS may be exaggerated.

## Conclusion

NCMS could remarkably increase the rural elderly’s reimbursement ratio but insignificantly promote the rural elderly’s OOP%. The proportion of reimbursement ratio for rural seniors with NCMS increased by 6.4% every 3 years. There may be several reasons for the gap between the nominal reimbursement ratio and actual reimbursement ratio and OOP%, which was not significantly reduced by the NCMS. These reasons may include lower outpatient reimbursement, migration, a limited reimbursement scope, an increasing demand for medical services and the rapid growth of medical expenses. Policymakers should further modify NCMS policies in rural China. Rural elderly people with relatively poor economic conditions and poor health levels have higher OOPs and lower reimbursement ratio. The government should implement corresponding medical assistance to prevent poverty among these people.

## Abbreviations

**CLHLS**: Chinese Longitudinal Healthy Longevity Survey
**CT**: Computed tomography
**MRI**: Magnetic resonance imaging
**NCMS**: New Cooperative Medical Scheme
**OOP**: out-of-pocket
**SUR**: seemingly unrelated regression
